# The In Vitro Enhancement of Retinal Cell Viability via m^6^A and m^5^C RNA Methylation-Mediated Changes in the Levels of Heme Oxygenase (HO-1) and DNA Damage Repair Molecules Using a 50 Hz Sinusoidal Electromagnetic Field (EMF)

**DOI:** 10.3390/ijms252413606

**Published:** 2024-12-19

**Authors:** Gabriela Betlej, Ewelina Bator, Anna Koziorowska, Marek Koziorowski, Iwona Rzeszutek

**Affiliations:** 1 Interdisciplinary Centre for Preclinical and Clinical Research, College of Natural Sciences, University of Rzeszow, Werynia 2a, 36-100 Kolbuszowa, Poland; 2 Institute of Material Engineering, College of Natural Sciences, University of Rzeszow, Pigonia 1, 35-310 Rzeszow, Poland; 3 Institute of Biotechnology, College of Natural Sciences, University of Rzeszow, Pigonia 1, 35-310 Rzeszow, Poland

**Keywords:** electromagnetic field (EMF), RNA methylation, heme oxygenase (HO), DNA damage repair, retina

## Abstract

Degenerative retinal diseases can lead to blindness if left untreated. At present, there are no curative therapies for retinal diseases. Therefore, effective treatment strategies for slowing the progression of retinal diseases and thus improving patients’ life standards are urgently needed. The present study aimed to assess the effect of sinusoidal electromagnetic field (EMF) (50 Hz, 1.3 mT) treatment for 15 and 30 min on spontaneously arising retinal pigment epithelial cells (ARPE-19) and retinal ganglion cells (RGC-5) and its short-term post-treatment significance. Our study indicated the beneficial impact of EMF treatment on the proliferative and migratory capacity of the tested cells. ARPE-19 and RGC-5 cells exposed to an EMF exhibited elevated levels of HO-1, increased N6-methyladenosine (m^6^A) and N5-methylcytosine (m^5^C) status mediated by METTL3 and NSUN2, respectively, and changes in levels of DNA damage repair factors, which may contribute to the regenerative properties of ARPE-19 and RGC-5 cells. Overall, this analysis showed that EMF (sinusoidal, 50 Hz, 1.3 mT) treatment may serve as a potential therapeutic strategy for retinal diseases.

## 1. Introduction

The retina is a continuation of the central nervous system (CNS) and is responsible for processing received visual information and transmitting it to the brain through the optic nerve [1]. It is a layered, complex structure composed of the retinal pigment epithelium (RPE), the rod and cone layer, the outer limiting layer, the outer nuclear layer (ONL), the outer plexiform layer (OPL), the inner nuclear layer (INL), the inner plexiform layer (IPL), the ganglion cell layer (GCL), the nerve fiber layer (NFL), and the inner limiting membrane [2]. The dysfunction or degeneration of the retina, such as age-related macular degeneration (AMD) or diabetic retinopathy (DR), are major causes of irreversible vision loss and blindness which impact the patients’ daily life [3]. Retinal diseases are correlated with inflammation, immunity, trauma, neurodegeneration, angiopathy, and other pathological conditions. Current treatment strategies involve laser photocoagulation, vitrectomy, repeated intravitreal injections of VEGF (anti-vascular endothelial growth factor) or steroids, and oral supplementation of antioxidants [4]. Although significant progress is being made in the field of retinal disorders, there are currently no curative treatments. Therefore, there is an urgent need to develop new approaches to manage these common and debilitating disorders.

Electromagnetic fields (EMFs) have been recently proposed to exert therapeutic potential [5,6,7,8,9]. The main advantage of EMF treatment over other treatment strategies is that it is non-invasive and can be applied in patients without anesthesia [10]. However, the effect of EMF treatment depends on the applied parameters, e.g., waveform, amplitude, frequency, time of exposure, as well as the model cell type and cell status [11]. Several studies have shown that the usage of EMF treatment may impact numerous biological processes through different mechanisms. The following mechanisms have been reported: changes in nitric oxide (NO) signaling [12,13], modulation of superoxide production [14,15], inhibition of apoptosis [16], and regulation of the inflammatory response [17], calcium homeostasis [18], and DNA damage repair signaling pathways. Recently, it has also been shown that EMF treatment may influence epitranscriptomic modification such as m^6^A RNA methylation [19].

Due to accumulating evidence of electromagnetic fields’ effectiveness in several clinical applications, we tested the impact of sinusoidal EMF (50 Hz, 1.3 mT) treatment on ARPE-19 and RGC-5 cells. We focused on the consequence of 15 min and 30 min exposition of ARPE-19 and RGC-5 cells to an EMF with a frequency of 50 Hz and intensity of 1.3 mT and examined its post-treatment effect. Changes in cell proliferation, viability, and migration, levels of heme oxygenase 1 (HO-1) and heme oxygenase 2 (HO-2), levels of METTL3 and NSUN2 methyltransferases, and m^6^A and m^5^C RNA methylation status, as well as levels of selected DNA damage response factors, were considered.

## 2. Results

### 2.1. Electromagnetic Field Treatment Stimulates Cell Viability, Proliferation, and Migratory Capacity in Retinal Cells In Vitro

**Figure 1:**
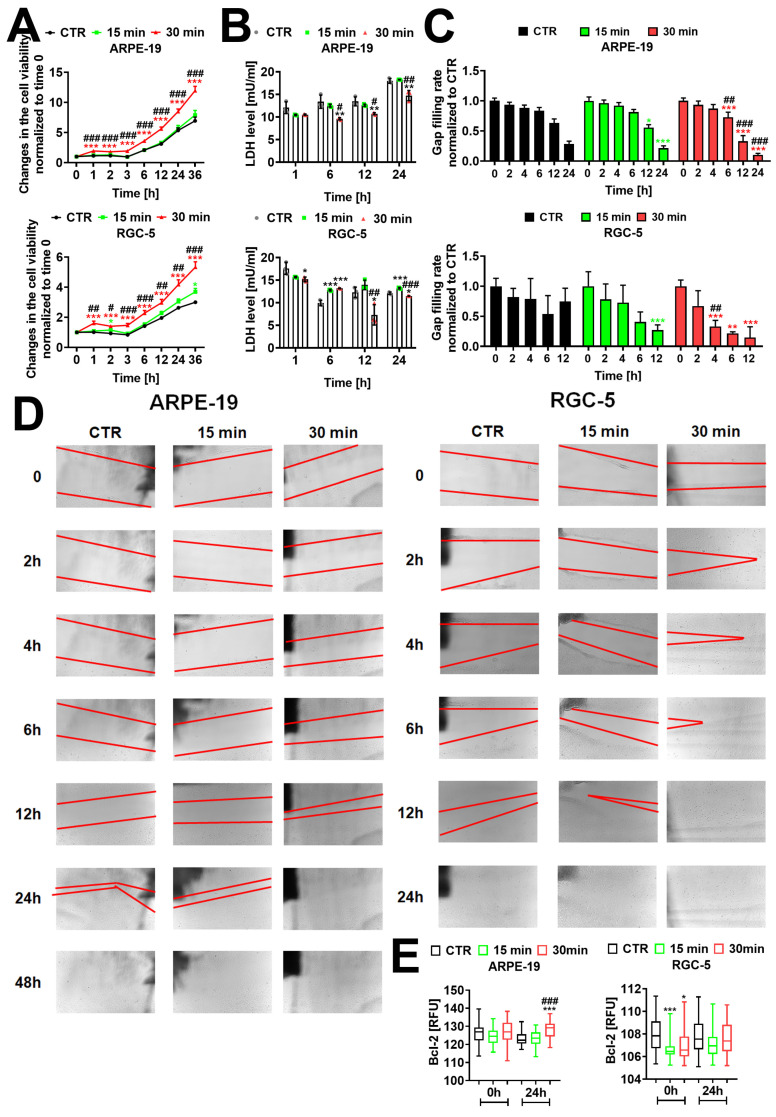
Electromagnetic field (EMF)-mediated changes in the ARPE-19 and RGC-5 cells’ fitness. (**A**) Changes in the cell viability of ARPE-19 and RGC-5 cells upon stimulation with the EMF. Cells were treated with a sinusoidal EMF (50 Hz; 1.3 mT) for 15 and 30 min and cell viability was analyzed at 0, 1, 2, 3, 6, 12, 24, and 36 h post-treatment using a RealTime-Glo™ MT Cell Viability Assay. Data were normalized to the 0 h time point. The line graphs indicate SD, *n* = 3 for each time point, *** *p* < 0.001, * *p* < 0.05, compared to the corresponding untreated control (CTR) (ANOVA and Dunnett’s a posteriori test), ^###^ *p* < 0.001, ^##^ *p* < 0.01, ^#^ *p* < 0.05 30 min of EMF treatment compared to 15 min of EMF treatment (one-way ANOVA and Tukey’s a posteriori test). (**B**) The levels of LDH were analyzed with a LDH-Glo™ Cytotoxicity Assay after 1, 6, 12, and 24 h of EMF treatment for 15 and 30 min and are presented as [mU/mL]. Bars indicate SD, *n* = 3, *** *p* < 0.001, ** *p* < 0.01, * *p* < 0.05 compared to the corresponding untreated control (CTR) (ANOVA and Dunnett’s a posteriori test), ^###^ *p* < 0.001, ^##^ *p* < 0.01, ^#^ *p* < 0.05 30 min of treatment compared to 15 min of EMF treatment (one-way ANOVA and Tukey’s a posteriori test). (**C**,**D**) The efficacy of wound healing of ARPE-19 and RGC-5 cells was analyzed up to 24 h post-treatment with the EMF for 15 and 30 min. Bars indicate SD, *n* = 12, *** *p* < 0.001, ** *p* < 0.01, * *p* < 0.05 compared to the corresponding untreated control (CTR) (ANOVA and Dunnett’s a posteriori test), ^###^ *p* < 0.001, ^##^ *p* < 0.01 30 min of treatment compared to 15 min of EMF treatment (one-way ANOVA and Tukey’s a posteriori test). Representative microphotographs are shown (**D**). Observations were made at 20× magnification. (**E**) Levels of Bcl-2 were detected using an immunostaining protocol and imaging cytometry. Results are presented as relative fluorescence units (RFU). Box and whisker plots are shown, *n* = 3, *** *p* < 0.001, * *p* < 0.05 compared to the corresponding untreated control (CTR) (ANOVA and Dunnett’s a posteriori test), ^###^ *p* < 0.001, 30 min EMF treatment compared to 15 min of EMF treatment (one-way ANOVA and Tukey’s a posteriori test). CTR—control conditions. EMF-treated cells—cells treated with 15 and 30 min of a sinusoidal electromagnetic field with a frequency of 50 Hz and intensity of 1.3 mT.

Electromagnetic field treatment has been shown to modulate cell viability and proliferation [18]. To investigate the effect of sinusoidal EMF (50 Hz and 1.3 mT) treatment on ARPE-19 and RGC-5 cells, a RealTime-Glo™ MT Cell Viability Assay was performed. A time course analysis indicated a significant increase in the cell viability of RGC-5 and ARPE-19 cells after 30 min of EMF treatment compared to the untreated CTR (*** *p* < 0.001, Figure 1A) and cells treated with 15 min of the EMF (^#^ *p* < 0.05, ^##^ *p* < 0.01, ^###^ *p* < 0.001, Figure 1A). Further, lactate dehydrogenase (LDH) levels at 1, 6, 12, and 24 h post-treatment with the EMF were also determined. We observed a significant decrease in the levels of LDH in ARPE-19 cells exposed to 30 min of the EMF after 6, 12, and 24 h compared to the untreated CTR (** *p* < 0.01, Figure 1B) and cells treated for 15 min (^#^ *p* < 0.05, ^##^ *p* < 0.01, Figure 1B). In RGC-5 cells, LDH levels significantly increased at the 6 h time point after 15 min and 30 min of exposition to the EMF (*** *p* < 0.001, Figure 1B). However, at 12 and 24 h post-treatment with the EMF for 30 min the LDH levels significantly decreased compared to the untreated CTR (* *p* < 0.05, Figure 1B) and cells treated for 15 min (^##^ *p* < 0.01, ^###^ *p* < 0.001, Figure 1B).

As the retinal pigment epithelium (RPE) cells surrounding the lesion migrate and proliferate to repair the wound [20], we tested whether the sinusoidal 50 Hz EMF treatment would influence cell migration and proliferation using an in vitro wound healing assay. A significant increase in cell migration/proliferation rate was observed in both cell lines 12 h post-treatment with 15 min of the EMF compared to CTR (* *p* < 0.05, *** *p* < 0.001, Figure 1C,D). Interestingly, accelerated cell migration/proliferation was observed already after 4 h and 6 h of 30 min of treatment in RGC-5 and ARPE-19 cells, respectively (** *p* < 0.01, *** *p* < 0.001, ^##^ *p* < 0.01, ^###^ *p* < 0.001, Figure 1C,D).

Chen et al. have shown that overexpression of Bcl-2 increases the survival of photoreceptors in mice with retinal degenerative disease, thus delaying its progression [21]. Therefore, we tested Bcl-2 levels in EMF-treated cells. Our analysis indicated a statistically significant increase in levels of Bcl-2 in ARPE-19 cells 24 h post-treatment with 30 min of the EMF compared to untreated CTR (*** *p* < 0.001, Figure 1E) and 15 min of treatment (^###^ *p* < 0.001, Figure 1E). Contrary, the levels of Bcl-2 were downregulated in RGC-5 cells right after the treatment with 15 min and 30 min of the EMF compared to CTR (* *p* < 0.05, ** *p* < 0.01, Figure 1E).

### 2.2. Electromagnetic Field (EMF) Treatment Modulates the Expression Levels of Heme Oxygenase 1 (HO-1) and Heme Oxygenase 2 (HO-2)

**Figure 2:**
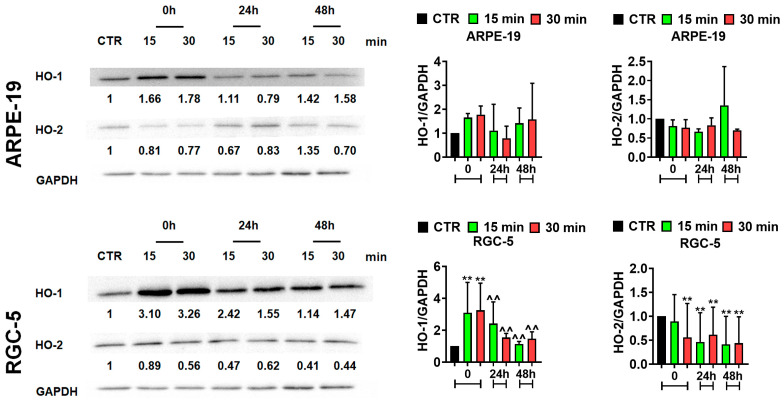
Electromagnetic field (EMF)-mediated changes in heme oxygenase 1 (HO-1) and heme oxygenase 2 (HO-2) levels in ARPE-19 and RGC-5 cells. Western blot-based analysis of HO-1 and HO-2 protein levels in CTR (untreated cells) and EMF-treated ARPE-19 and RGC-5 cells. The effect of EMF treatment on HO-1 and HO-2 protein levels was assessed at the 0 h, 24 h, and 48 h time points. Anti-GAPDH served as a loading control. Data were normalized to GAPDH. Bars indicate SD, *n* ≥ 2 ** *p* < 0.01 compared to the corresponding untreated control (CTR) (ANOVA and Dunnett’s a posteriori test), ^^^^ *p* < 0.01 compared to the corresponding 0 h time point (one-way ANOVA and Tukey’s a posteriori test). CTR—control conditions. EMF-treated cells—cells treated with 15 and 30 min of a sinusoidal electromagnetic field with a frequency of 50 Hz and intensity of 1.3 mT.

Heme oxygenase 1 (HO-1) and heme oxygenase 2 (HO-2) share the same mechanism of action and convert heme to biliverdin (BV) [22]. HO-1 (inducible form) and HO-2 (constitutive form) possess antioxidant, cytoprotective, anti-inflammatory, and neurotransmitter properties [23,24,25]. Therefore, we determined the effect of the sinusoidal EMF (50 Hz, 1.3 mT) on HO-1 and HO-2 protein levels in RGC-5 and ARPE-19 cells (Figure 2). Interestingly, the levels of HO-1 increased right after 15 min and 30 min of treatment with the EMF in ARPE-19 and RGC-5 cells compared to CTR; however, the observed elevation of the HO-1 levels was statistically significant only in RGC-5 cells (** *p* < 0.01, Figure 2). The levels of HO-1 stayed slightly up-regulated for 48 h post-treatment, except at the 24 h time point where the ARPE-19 cells were treated for 30 min with the EMF. In RGC-5 cells, the levels of HO-1 significantly decreased with the time of post-treatment incubation compared to its corresponding 0 h time point (^^ *p* < 0.01, Figure 2). Conversely, the levels of HO-2 stayed downregulated for 48 h post-treatment in ARPE-19 and RGC-5 cells treated with 15 min and 30 min of the EMF compared to untreated CTR, except the 48 h time point, where in ARPE-19 cells treated for 15 min with the EMF, the levels of HO-2 suddenly increased compared to CTR. However, the observed changes in the HO-2 levels were statistically significant in RGC-5 cells (** *p* < 0.01, Figure 2). These results showed that EMF treatment stimulates HO-1 levels in retinal cells.

### 2.3. Changes in m^5^C and m^6^A Status in Electromagnetic Field (EMF)-Exposed ARPE-19 and RGC-5 Cells

**Figure 3:**
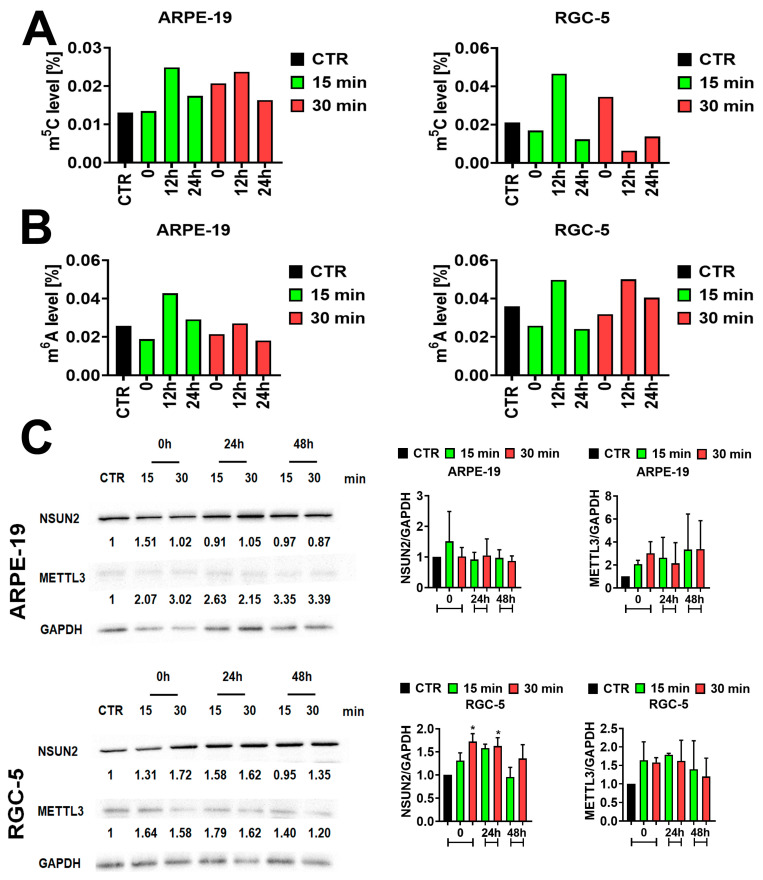
Analysis of m^6^A and m^5^C RNA methylation status and protein levels of the RNA methyltransferases METTL3 and NSUN2 in CTR and EMF-treated ARPE-19 and RGC-5 cells. (**A**) ELISA-based assessment of RNA:m^5^C methylation levels in CTR and EMF-treated ARPE-19 and RGC-5 cells, presented as [%]. Analysis of RNA:m^5^C methylation levels in cells exposed to EMF treatment for 15 min and 30 min were made at the 0 h, 12 h, and 24 h time points. Untreated cells served as a control (CTR). Bars indicate SD, *n* = 2. (**B**) Analysis of RNA:m^6^A methylation levels [%] in CTR and EMF-treated ARPE-19 and RGC-5 cells using ELISA. Analysis of RNA:m^6^A methylation levels in cells exposed to EMF treatment for 15 min and 30 min were made at the 0 h, 12 h, and 24 h time points. Untreated cells served as a control (CTR). Bars indicate SD = 2. (**C**) Western blot-based analysis of protein levels of the METTL3 and NSUN2 methyltransferases in CTR and EMF-treated ARPE-19 and RGC-5 cells. The effect of EMF treatment on METTL3 and NSUN2 protein levels was measured at the 0 h, 24 h, and 48 h time points. The anti-GAPDH antibody served as a loading control. Data were normalized to GAPDH. Bars indicate SD, *n* ≥ 2, * *p* < 0.05 compared to the corresponding untreated control (CTR) (ANOVA and Dunnett’s a posteriori test). CTR—control conditions (untreated cells). EMF-treated cells—cells treated with 15 and 30 min of a sinusoidal electromagnetic field with a frequency of 50 Hz and intensity of 1.3 mT.

RNA modifications including N5-methylcytosine and N6-methyladenosine are installed by responsible enzymes called methyltransferases [26]. NOP2/Sun RNA methyltransferase family member 2 (NSUN2) mediates RNA m^5^C methylation [27], while methyltransferase-like 3 (METTL3) is an S-adenosylmethionine-(SAM-) binding protein responsible for m^6^A methylation [28]. These post-transcriptional modifications may influence many cellular processes, namely cell death, metabolism, proliferation, differentiation, migration, senescence, autophagy, and the DNA damage response [29]. Currently, several approaches are used to evaluate RNA methylation, each possessing its own drawbacks [30]. Our choice was an ELISA-based assay, where the intensity of the signal originating from the RNA sample is compared to the standard curve obtained from known nonmethylated and methylated RNA to analyze the extent of methylation in the tested sample. The major disadvantage of this technique is that it is not possible to predict which transcript is affected by differential methylation as total RNA is used for analysis. However, according to the manufacturer, the kit is sensitive, specific, accurate, and universal. Our analyses indicated that 15 min of EMF treatment stimulates an increase of m^5^C and m^6^A levels, peaking at 12 h, and decreasing 24 h post-treatment in ARPE-19 and RGC-5 cells (Figure 3A,B). A similar effect was observed in the case of m^5^C and m^6^A methylation after 30 min of EMF treatment in ARPE-19 and RGC-5 cells, respectively. Conversely, 30 min of EMF treatment stimulated the highest m^5^C levels at the basal time point (0 h), which decreased with the time of incubation in RGC-5 cells. The m^6^A levels present in ARPE-19 cells after 30 min of EMF treatment were comparable to those in untreated cells (Figure 3B).

Subsequently, we tested the levels of enzymes responsible for those modifications, namely NSUN2 and METTL3 (Figure 3C). ARPE-19 cells exposed to 15 min and 30 min of the sinusoidal EMF did not show significant changes in NSUN2 levels compared to the control. The exposure of RGC-5 cells to 15 and 30 min of EMF treatment stimulated the levels of NSUN2 methyltransferase compared to CTR; however, only 30 min of treatment caused a statistically significant elevation of NSUN2 levels (* *p* < 0.05, Figure 3C). NSUN2 levels stayed up-regulated 24 h post-treatment with 15 min of the EMF compared to CTR and the 0 h time point and next decreased to the levels of the untreated CTR. In RGC-5 cells exposed to 30 min of the EMF, NSUN2 levels were significantly elevated 24 h post-treatment compared to CTR (untreated cells) (* *p* < 0.05, Figure 3C). In ARPE-19 and RGC-5 cells, the levels of METTL3 increased after 15 and 30 min of EMF treatment compared to the untreated control. In ARPE-19 cells exposed to 15 min of the EMF, the levels of METTL3 increased with the time of post-treatment incubation. Conversely, 30 min of EMF treatment caused the downregulation of METTL3 levels observed 24 h post-treatment compared to the 0 h time point. Interestingly, in RGC-5 cells, METTL3 levels reached the highest peak 24 h post-treatment and next decreased, regardless of the time of EMF exposition. Although EMF treatment influenced METTL3 levels in ARPE-19 and RGC-5 cells, the observed changes were not prominent enough to be statistically significant.

### 2.4. The Effect of Electromagnetic Field (EMF) Treatment on DNA Damage Response (DDR) in ARPE-19 and RGC-5 Cells

**Figure 4:**
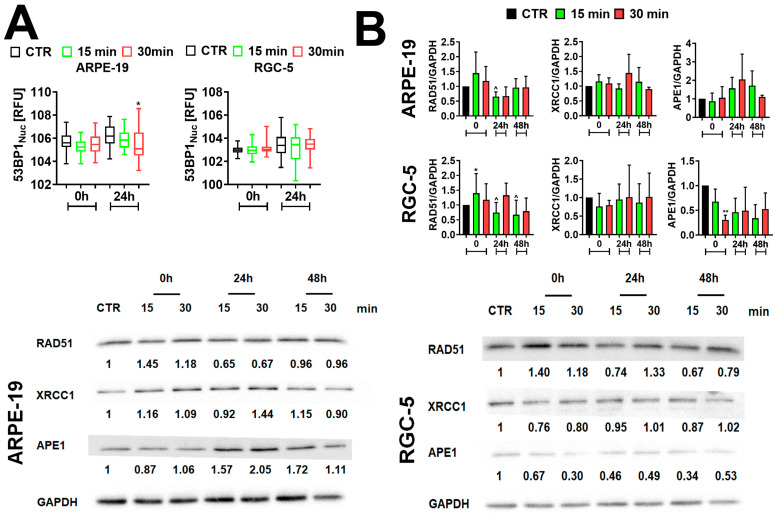
Electromagnetic field (EMF)-mediated DNA damage response in ARPE-19 and RGC-5 cells. (**A**) Immunostaining- and imaging cytometry-based 53BP1 levels. Results are presented as relative fluorescence units (RFU). Box and whisker plots are shown, *n* = 3, * *p* < 0.05 compared to the corresponding untreated control (CTR) (ANOVA and Dunnett’s a posteriori test). (**B**) Western blot-based analysis of the levels of proteins involved in DNA damage repair in ARPE-19 and RGC-5 cells. Anti-GAPDH served as a loading control. Data were normalized to GAPDH. Bars indicate SD, *n* ≥ 2, ** *p* < 0.01, * *p* < 0.05 compared to the corresponding untreated control (CTR) (ANOVA and Dunnett’s a posteriori test), ^^^ *p* < 0.05 compared to the corresponding 0 h time point (one-way ANOVA and Tukey’s a posteriori test). CTR—control conditions, untreated cells. EMF-treated cells—cells treated with 15 and 30 min of a sinusoidal electromagnetic field with a frequency of 50 Hz and intensity of 1.3 mT and analyzed at 0 h, 24 h, and 48 h post-treatment.

It has been shown that the overexpression of Bcl-2 may aid DNA repair after oxidative stress [31] and promotes the growth and regeneration of retinal axons in the central nervous system (CNS) [32]. Thus, this may suggest that the elevated levels of Bcl-2 present in ARPE-19 cells observed 24 h post-treatment with 30 min of the EMF may stimulate the DNA repair machinery, hence contributing to the cells’ regeneration. Thereby, we examined the levels of different components (e.g., 53BP1, RAD51, XRCC1, and APE1) of DNA repair pathways in EMF-treated ARPE-19 and RGC-5 cells. The levels of 53BP1 did not significantly change upon EMF treatment compared to CTR, except in the ARPE-19 cells where we observed downregulation of 53BP1 levels 24 h post-treatment with 30 min of the EMF (* *p* < 0.05, Figure 4A). Additionally, our study demonstrated that 15 min and 30 min of EMF treatment stimulated the levels of RAD51 in ARPE-19 and RGC-5 cells at the 0 h time point compared to the untreated CTR. However, only 15 min of EMF treatment had a statistically significant effect on RGC-5 cells (* *p* < 0.05, Figure 4B). In the case of 15 min of EMF exposition, the levels of RAD51 decreased already at 24 h post-treatment compared to the untreated CTR and at the 0 h time point in ARPE-19 and RGC-5 cells (^ *p* < 0.05, Figure 4B). A similar effect was observed after 30 min of treatment in ARPE-19 cells. Conversely, in RGC-5 cells treated with the EMF for 30 min, the levels of RAD51 stayed up-regulated up to 24 h post-treatment, and next decreased. The expression of XRCC1 did not significantly change in ARPE-19 cells after 15 min and 30 min treatment with the EMF compared to CTR. Exposition to 15 min and 30 min of the EMF caused downregulation of APE1 levels in RGC-5 cells compared to the untreated CTR; however, only 30 min of exposition to the EMF caused a statistically significant effect (** *p* < 0.01, Figure 4B). APE1 levels stayed downregulated for 48 h post-treatment in RGC-5 cells compared to CTR. In ARPE-19 cells, the levels of APE1 increased 24 h and 48 h post-treatment in cells treated with 15 min of the EMF compared to CTR. A two-fold increase of APE1 levels was observed in ARPE-19 cells 24 h after 30 min of EMF treatment. Although the EMF influenced the levels of APE1, the changes were not statistically significant.

## 3. Discussion

Our investigation of the effect of a sinusoidal electromagnetic field (EMF) on retinal cells (ARPE-19 and RGC-5) revealed the beneficial impact of 30 min of exposition to an EMF with a frequency of 50 Hz and maximal induction of 1.3 mT on cell viability and migration/proliferation. ARPE-19 cells exhibited higher proliferative capacity after EMF treatment compared to RGC-5 cells; however, this is not surprising as ganglion cells are not as metabolically active as retinal pigment epithelial cells [33]. The obtained data are consistent with our previous studies showing that an EMF (50 Hz and 2–6 mT) stimulates the cell viability of noncancerous BJ and HEK cells [34]. Additionally, Huo et al. have shown that a non-invasive EMF stimulates the migration of keratinocytes and fibroblasts as well as serves as a weak proliferation promoter of keratinocytes [35]. Exposure of HaCaT cells to an EMF with a frequency of 50 Hz and maximal induction of 1 mT has been correlated with a positive effect on cell proliferation [36,37]. Patruno et al. found that the molecular mechanism responsible for this phenomenon involves the modulation of the activation of mTOR (PI3K/Akt) and ERK signaling pathways [36]. Subsequently, the same group has shown that an EMF has the ability to modulate inflammation factors and thus plays an important role in wound healing [38]. The positive effect of EMFs has also been confirmed in vivo where faster wound healing was observed after treatment with a pulsed EMF (30 Hz and 0.8 T) for 30 min daily within 3 weeks, as compared to the control group [39]. Additionally, it has been shown that a pulsed EMF with frequencies and intensities within ranges of less than 100 Hz and 3 mT, respectively, correlates with an increased efficiency of the wound healing process [40,41]. Subsequent animal studies have demonstrated that a pulsed EMF has a positive effect on wound size reduction in comparison to the control group [42,43]. Also, human clinical studies have shown that EMF accelerates healing time and reduce the rate of recurrence of venous leg ulcers [44,45]. Therefore, our analyses are in agreement with the presented studies and suggests stimulatory properties of sinusoidal EMF (50 Hz, 1.3 mT) on retinal cell viability and growth, which are essential for cell regeneration.

Oxidative stress is one of the causes of retinal diseases [46]. Therefore, we investigated whether the tested conditions of an EMF may stimulate antioxidative enzymes, thus contributing to a curing effect in retinal cells. To test this, we analyzed the levels of two isoforms of heme oxygenase, namely heme oxygenase-1 (HO-1) and heme oxygenase-2 (HO-2). HO-1 and HO-2 are enzymes that catabolize heme to generate biliverdin (BV), ferrous iron, and carbon monoxide (CO), and possess an anti-inflammatory, antiapoptotic, and immunomodulatory effect [22]. Our data indicated elevated levels of HO-1 after EMF treatment which stayed up-regulated 48 h post-treatment in ARPE-19 and RGC-5 cells compared to the untreated CTR. Contrary, the exposure to the EMF led to the downregulation of HO-2 levels compared to CTR. Interestingly, the EMF treatment exerted a more prominent effect on changes in HO-1 and HO-2 levels in RGC-5 compared to ARPE-19 cells. The downregulation of HO-2 expression is not surprising as its diminution has been already correlated with HO-1 up-regulation in human cell lines [47]. Interestingly, a positive role of HO-1 in retinal endothelial cells has been already documented. For instance, HO-1 has been shown to exert a protective function toward retinal endothelial cells exposed to high-glucose and oxidative/nitrosative stress conditions [48]. The beneficial effects of HO-1 and its metabolites have been reported in the rat retina, where it promoted cell survival after retinal ischemia-reperfusion injury [49]. Hmox1 overexpression has also been shown to attenuate neuronal cell death caused by oxidative stress in mouse astrocytes [50]. Furthermore, HO-1 induced pharmacologically enhanced proliferation and migration in human microvascular endothelial cells in vitro [51,52]. Moreover, the significance of HO-1 has been demonstrated in wound healing and retinal ischemia models in vivo [53]. One may argue that EMFs may cause ROS production, thereby activating the antioxidant properties of HO-1. However, controlled oxidative stress has been reported as a positive sign of tissue repair and regeneration [54]. Overall, our data suggests that HO-1 expression contributes to the increased proliferative and migratory capacity of EMF-treated ARPE-19 and RGC-5 cells.

Interestingly, Chan et al. indicated that yeast exposed to H_2_O_2_ were capable of selective translation of mRNA coding for proteins required for the oxidative stress response through elevated levels of m^5^C at the wobble position of tRNA^Leu(CAA)^. Similarly, elevated levels of m^6^A in tRNA^Val^ present in *E. coli* influenced growth advantage under oxidative stress conditions [55]. Additionally, RNA m^6^A methylation has been implicated in the ability of skin to self-renew and heal wounds [56] and cells’ regenerative capacity [57]. m^5^C modification has also contributed to the proper cell proliferation and migration of HEK293 cells [58]. Recently, it has been shown that the loss of m^6^A modification and downregulation of METTL3 expression correlates with liver, heart, and skeletal muscle aging and thus age-related phenotypes [59]. Therefore, we wanted to know whether elevated levels of antioxidative HO-1 are related to the changes in the RNA methylation status in EMF-exposed cells and thereby contribute to cell proliferative properties. In this study, ARPE-19 and RGC-5 cells treated with an EMF presented an increase in their levels of m^6^A and m^5^C modifications compared to CTR. The levels of methyltransferases (METTL3 and NSUN2) responsible for the studied RNA modifications were also up-regulated. However, the levels of NSUN2 were more evident in RGC-5 cells. Interestingly, Nsun2−/− mice and human skin cells exhibited an increase in apoptotic cell death in response to sodium arsenite- or UV-induced oxidative stress, which corresponded with the diminution of NSUN2-mediated RNA methylation [60]. Therefore, observed changes in RNA methylation status may be linked with ARPE-19 and RGC-5 cells’ proliferative and migratory capacities, thus contributing to their regenerative properties. This statement is supported by studies performed in vivo showing a significant role of METTL3-mediated m^6^A modification in muscle stem cells and muscle regeneration [61].

Recently, the involvement of DNA replication and repair factors has been correlated with the large-scale tissue regenerative process [62]. Therefore, we analyzed the levels of RAD51, XRCC1, and APE1 in ARPE-19 and RGC-5 cells treated with an EMF. Our analysis indicated that the exposition to the EMF modified the expression of the tested factors. Interestingly, the EMF facilitated the expression of RAD51 at the 0 h time point, which decreased 24 h post-treatment. Indeed, recent studies have demonstrated that animals lacking Rad51 fail to regenerate cells [63]. Additionally, other studies have shown that during the regeneration process, DDR-related genes are mostly up-regulated. Analyses performed on regenerative tissues indicated the up-regulation of DNA repair genes across multiple organs and animal species, suggesting a pivotal role of DDR in regenerative mechanisms [62,64,65].

## 4. Materials and Methods

### 4.1. Cell Culture

Human retinal pigment epithelial cells (ARPE-19; cat. no: CRL-2302) and mouse retinal ganglion cells (RGC-5; cat. no: PTA6600) were purchased from The American Type Culture Collection (ATCC). Cells were cultured under standard conditions (at 37 °C in the presence of 5% CO_2_) in DMEM/Ham’s F12 medium (ARPE-19) or DMEM (RGC-5) (Capricorn Scientific, Ebsdorfergrund, Germany) supplemented with 10% FBS and 100 U/mL penicillin, 0.1 mg/mL streptomycin, and 0.25 μg/mL amphotericin B (Corning, Camelback Rd. Glendale, CA, USA).

### 4.2. EMF Treatment

Cells were exposed to a sinusoidal continuous 50 Hz electromagnetic field at 1.3 mT for 15 and 30 min in culture dishes placed inside the solenoid. After treatment, cells were incubated up to 48 h to test for post-treatment effects. Untreated cells served as the CTR. The experimental conditions were selected based on the literature [66,67]. The sinusoidal EMF was generated using a µPulse 10 placed into a cell culture incubator, Cell Expert C170 (Eppendorf, Hamburg, Germany). The parameters of the electromagnetic field were controlled by a sensor integrated into the system. The temperature of the plates was controlled by a sensor mounted in the metal plate under the tested plate, while the parameters required for the cells’ growth were provided by the cell culture incubator.

### 4.3. Cell Viability Analysis

To monitor cell viability in real time after EMF treatment, a RealTime-Glo™ MT Cell Viability Assay (Promega, Walldorf, Germany) was performed. In brief, cells seeded onto 96-well plate were exposed to the EMF field as it was described previously. Next, RealTime-Glo™ MT mix was added to the medium and luminescence was measured at 0, 1, 2, 3, 6, 12, 24, and 36 h post-treatment using an Infinity M200 PRO microplate reader (Tecan, Männedorf, Switzerland).

### 4.4. Determination of Lactate Dehydrogenase (LDH) Concentration

LDH levels in cell medium in response to electromagnetic field treatment were determined using an LDH-Glo™ Cytotoxicity Assay (Promega). Cell culture medium was collected 1, 6, 12, and 24 h after EMF treatment for 15 and 30 min and stored at −20 °C in Storage Buffer. Untreated cells were used as a control (CTR). On the day of analysis, all procedure steps were performed according to the manufacturer’s instructions. Luminescence detection was performed using an Infinity M200 PRO microplate reader (Tecan, Männedorf, Switzerland). To avoid false positive results due to differences when implanting cells into culture wells, the results were calculated in terms of the luminescence emitted by the cells at the time point 0 h and normalized.

### 4.5. Wound Healing Assay

Cells were seeded onto 6-well plates and grown to 90%. Cells were treated by EMF for 15 and 30 min and wounded gaps across the cells were created using sterile pipette tips. After washing the wound gaps with PBS, images were captured at different time points between 0 and 24 h during culture, using an Olympus IX73. The migration/proliferation ratio was calculated using OLYMPUS cellSens Standard Software, Ver. 3.1.1. Due to differences in the sizes of the scratches, the results were calculated and normalized to the results obtained at time 0 h for each of the analyzed variants.

### 4.6. RNA Isolation and Measurement of the Levels of m^5^C and m^6^A in RNA

RNA was isolated from cell culture using a Total RNA Maxi kit (A&A Biotechnology, Gdansk, Poland). The procedure was performed according to the manufacturer’s recommendation. Isolated RNA was measured and stored at −86 °C for future procedures.

The levels of m^5^C and m^6^A were quantified using fluorometric and colorimetric commercially available ELISA kits (MethylFlash 5-mC RNA Methylation ELISA Easy Kit and EpiQuik m6A RNA Methylation Quantification Kit; EpigenTek, Farmingdale, NY, USA), respectively. On the day of analysis, RNA was diluted to the desirable concentration and all steps were performed according to the manufacturer’s instructions. The levels of m^5^C and m^6^A in RNA were determined using an Infinity M200 PRO (Tecan, Männedorf, Switzerland).

### 4.7. Homogenate Preparation and Western Blotting

Cells were seeded onto 6-well plates. On the next day, cells were treated with an EMF as was described previously. Cells were collected from the plate and protein was isolated using RIPA buffer. Protein concentration was measured using the BCA method (Thermo Fisher Scientific, Waltham, MA, USA). Next, protein samples were separated in SDS–PAGE and transferred onto PVDF membranes (Thermo Fisher Scientific, Waltham, MA, USA). Membranes were blocked in 1% BSA for 1 h and incubated with primary antibodies (anti-APE1 BT-AP07734, 1:1000; anti-NSUN2 BT-AP11581, 1:1000; anti-METTL3 RA20420, 1:1000; anti-XRCC1 MA5-13412, 1:400; anti-RAD51 PA5-27195, 1:1000; anti-HO-1 MA5-45152, 1:1000; anti-HO-2 PA5-28334, 1:1000; anti-GAPDH MA1-16757, 1:2000) and secondary horseradish peroxidase (HRP)-conjugated antibodies (#31430 or #31460, 1:10,000). Detection was performed using an enhanced chemiluminescence (ECL) substrate (Thermo Fisher Scientific, Waltham, MA, USA). The relative protein level was normalized to GAPDH concentration using ImageJ software (https://imagej.nih.gov/ij/, U. S. National Institutes of Health, Bethesda, MD, USA).

### 4.8. Imaging Cytometry

After EMF treatment, ARPE-19 and RGC-5 cells were fixed and subjected to an immunostaining protocol as previously described [68]. The cells were incubated with primary antibodies, Bcl-2 (1:50, SAB5700155, Merck, Schnelldorf, Germany), and 53BP1 (1:1000, PA1-16565, Thermo Fisher Scientific, Waltham, MA, USA) at 4 °C overnight. Next, the secondary antibodies conjugated to Texas Red (1:1000, T2767) or FITC (1:1000, F2761) (Thermo Fisher Scientific, Waltham, MA, USA) were applied at room temperature for 1 h. Nuclei were stained using Hoechst 33342. Cell images were acquired using an IN Cell Analyzer 2000 confocal imaging system (GE Healthcare Life Sciences, Piscataway, NJ, USA). The immunofluorescent signals of the protein levels are presented as relative fluorescent units (RFUs).

### 4.9. Statistical Analysis

All experiments were performed with at least three independent repetitions. The results represent the mean ± SD. Differences between the control and treated samples were analyzed using a one-way ANOVA and Dunnett’s test, whereas differences between samples were calculated using a one-way ANOVA and Tukey’s multiple comparison test. Statistical significance was assessed using GraphPad Prism 8. *p*-values of less than 0.05 were considered significant.

## 5. Conclusions

**Figure 5:**
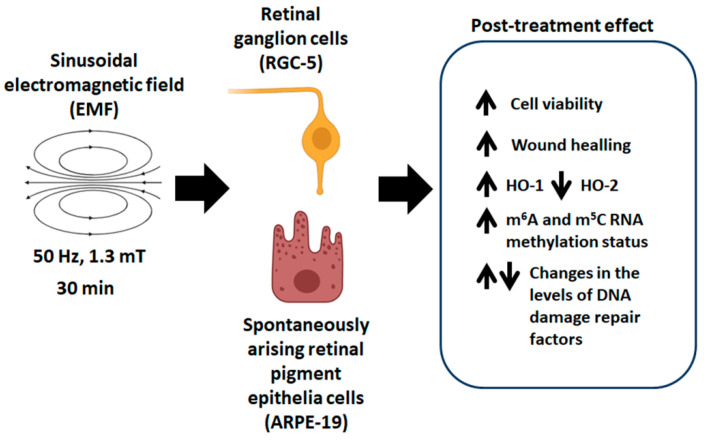
Scheme showing the effect of the sinusoidal electromagnetic field (EMF) with an induction of 50 Hz and frequency of 1.3 mT on ARPE-19 and RGC-5 cells. Upward arrows (↑) indicate an up-regulation, while downward arrows (↓) indicate a down-regulation.

In this study, we established the effect of a sinusoidal 50 Hz electromagnetic field (EMF) on retinal cells, namely ARPE-19 and RGC-5 cells (Figure 5). We showed that after 30 min of exposition to a sinusoidal EMF with a frequency of 50 Hz and intensity of 1.3 mT, cells become activated, and in this state, they change their proliferative and migration capacity. We suggest that this effect is due to a boost of heme oxygenase 1 (HO-1) levels and modulation of expression of the DNA damage repair factors mediated by transient elevation of m^5^C and m^6^A methylation status. Overall, we suggest that the sinusoidal 50 Hz EMF has a beneficial effect on retinal cell migration and proliferation and may serve as a potential tool for the treatment of retinal diseases. 

## Data Availability

The raw data supporting the conclusions of this article will be made available by the authors on request.
